# Unsupervised Assessment of Balance and Falls Risk Using a Smartphone and Machine Learning

**DOI:** 10.3390/s21144770

**Published:** 2021-07-13

**Authors:** Barry R. Greene, Killian McManus, Lilian Genaro Motti Ader, Brian Caulfield

**Affiliations:** 1 Kinesis Health Technologies, D04 V2N9 Dublin, Ireland; killian.mcmanus@kinesis.ie; 2 Insight Centre, University College Dublin, D04 N2E5 Dublin, Ireland; b.caulfield@ucd.ie; 3 Department Computer Science and Information Systems, University of Limerick, V94 XT66 Limerick, Ireland; lilian.mottiader@ul.ie

**Keywords:** falls, balance, postural sway, smartphone, inertial sensor, accelerometer, gyroscope

## Abstract

Assessment of health and physical function using smartphones (mHealth) has enormous potential due to the ubiquity of smartphones and their potential to provide low cost, scalable access to care as well as frequent, objective measurements, outside of clinical environments. Validation of the algorithms and outcome measures used by mHealth apps is of paramount importance, as poorly validated apps have been found to be harmful to patients. Falls are a complex, common and costly problem in the older adult population. Deficits in balance and postural control are strongly associated with falls risk. Assessment of balance and falls risk using a validated smartphone app may lessen the need for clinical assessments which can be expensive, requiring non-portable equipment and specialist expertise. This study reports results for the real-world deployment of a smartphone app for self-directed, unsupervised assessment of balance and falls risk. The app relies on a previously validated algorithm for assessment of balance and falls risk; the outcome measures employed were trained prior to deployment on an independent data set. Results for a sample of 594 smartphone assessments from 147 unique phones show a strong association between self-reported falls history and the falls risk and balance impairment scores produced by the app, suggesting they may be clinically useful outcome measures. In addition, analysis of the quantitative balance features produced seems to suggest that unsupervised, self-directed assessment of balance in the home is feasible.

## 1. Introduction

An estimated 30% of adults over the age of 65 fall each year, with 1 in 5 dying within 12 months. 1 in 3 of those experiencing a fall never return home after a fall-related hip fracture [1]. The direct healthcare cost of falls has been estimated to be $50 Bn in the US [2] and €25 Bn per annum in the EU [3]; an ageing population means that these costs are increasing steadily each year. Falls are not inevitable and research has clearly shown that the incidence of falls can be reduced by up to 50% through early intervention [4,5]. However, effective early intervention is dependent on accurate screening and identification of those people at risk of experiencing a fall, before the first fall has occurred, ensuring appropriate referral for patients at higher risk [6]. Self-directed, remote assessment of falls risk prior to the first fall, would allow timely intervention and ease the burden on overstretched healthcare systems [6].

The past two decades have seen an increased democratization in our capacity to use digital tools to measure human behaviour and performance. Traditionally, there was a compromise between relying on either clinic-based observational measures that are subjective in nature or laboratory based measurements that are complex, expensive and available to a very small minority of specialists.

In recent years, the ubiquity and technical sophistication of wearables and smartphones has led to a sharp increase in the availability of mobile apps and body-worn devices for health monitoring and assessment. This has facilitated a move towards clinic and field based measures, greatly increasing our capacity to objectively capture human performance for clinical and research applications. Though a significant advance, this approach still requires a clinician or investigator “in-the-loop” to ensure appropriate adherence to the test protocol and thus limits scalability in a clinical research or public health context.

More recently, we have seen the development of a range of wearable and mHealth technologies that seek to transfer the responsibility for data capture (as well as performance measurement) to the patient or end user [7]. This trend has accelerated due to the COVID-19 pandemic, with health systems under pressure and vulnerable older people at heightened risk of infection. This approach has huge potential as it promises true scalability. Clinical trials could be augmented by means of patient-led data capture in the home, while community risk screening programs could rely on frequent measures captured by people in their own homes. However, verifying the provenance and clinical value of data captured unsupervised by the patient in the home setting has become increasingly important, given the potential harms arising from improperly validated apps [8,9].

One area where a patient-led data capture approach has gained some traction is in the measurement of balance and postural control. Leveraging the inertial sensors incorporated into most smartphones is a powerful model, given that 92% of over 65s in the USA have a mobile phone (with 62% owning a smartphone) [10], and therefore have the ability to capture ongoing data related to their balance and falls risk. This approach offers the potential to implement large scale community-sourced screening programs for major public health issues such as falls in older adults.

In the context of a digital approach to prevent falls, the use of technologies for patient-led data capture, such as balance and falls risk assessment, is fundamental to enable personalised interventions, encouraging users to adopt a routine of strength and balance exercises [11,12]. Patient-led data capture supports tailored rehabilitation programmes in the home, given that self-assessment has the potential to be used to define the exercise protocol (usually requiring a physiotherapist [13]), as well as to monitor progress [14]. A number of systematic reviews of the literature have examined existing applications for balance and falls risk assessment, evaluating a variety of functional tasks, and sensing devices [11,12,15,16,17,18]. However, mHealth applications should be designed with special consideration given to the heterogeneity of the older adult’s needs and skills, in order to make sure users can properly operate the system [18] and perform the task safely [19].

Inertial sensors (usually embedded in the current generation of smartphones), have been demonstrated to allow reliable and accurate measurement of postural sway [20,21,22,23]. The challenge lies in demonstrating that community-sourced, patient-led, unsupervised measurement of balance using a smartphone app could yield data of sufficiently high quality for use in driving remedial interventions.

In this study we will address this issue using a dataset acquired during the initial phase of deployment of a smartphone application that uses a self-directed measurement of standing balance alongside a questionnaire, capturing self-reported data related to falls history and clinical risk factors. The objective was to determine whether biomarkers derived from questionnaire responses and self-directed, unsupervised balance measurements were related to self-reported risk factors. We report an observational study of the performance of a smartphone application for assessment, management and prevention of falls risk in older adults, tested on a large statistically independent sample. We also compare the quantitative balance features obtained from the smartphone sensors against equivalent measures obtained using a lumbar sensor from an independent reference data set.

The object of the present study, a smartphone application for assessment, management and prevention of falls risk in older adults, contains algorithms for prediction of falls and assessment of balance that have been developed, trained and validated over the past 13 years. A number of apps for assessment of balance or prevention of falls have been reported in the literature, but few report the validity of a smartphone for assessment of balance and falls risk. However, a review of the literature shows that there is a need for reliable and accurate unsupervised assessment [19]. We believe the present study is the first to examine how algorithms trained to assess balance and falls risk on an independent dataset, using lumbar IMU data obtained during standing balance tests, behave when deployed on a smartphone unsupervised and self-directed ‘in the wild’.

## 2. Data

### 2.1. Smartphone App Data

This study reports an analysis of anonymised data obtained from users of the Kinesis Balance™ fall prevention app (Kinesis Health Technologies, Dublin, Ireland), a Class I medical device, between September 2020 and May 2021. The app is freely available on the Google Play store, with participants consenting to use of their anonymised data for research and product improvement. The app uses a questionnaire on clinical falls risk factors, combined with a standing balance test and machine learning algorithms to assess balance and falls risk. Participants are provided with advice and exercises to stay healthy and help prevent falls, based on their level of risk (as determined by the smartphone based machine learning algorithm). All data were anonymised at source and transmitted via encrypted channel to a web server where they were stored (in line with GDPR and HIPAA data privacy regulations) for subsequent offline analysis. Data were extracted from the database using MySQL workbench 8.0 (Oracle, CA, USA) and analysed using Matlab v9.3.0 (Mathworks, Natick, VA, USA).

### 2.2. Reference Data Set

A reference data set [24,25] is embedded within the app and was used to compare against the smartphone inertial sensor data. The reference data consist of 277 (178 female, age 74.7 ± 6.6 years) participants; each participants had their balance assessed in a clinical environment, using a standing balance test with an inertial sensor (Kinesis Health Technologies, Dublin, Ireland) mounted on their lower back (attached to L3 lumbar spine with double-sided tape) and sampled at 102.4 Hz. Each participant completed the balance test by standing still for 30 s with eyes open (in a semi-tandem stance) and eyes closed (in a narrow stance). Clinical fall risk factors were captured for each subject by means of assessment using a Comprehensive Geriatric Assessment and the Berg Balance Scale; while 100 of 277 participants reported a history of falls in the previous 12 months.

## 3. Methods

Upon installing the app, participants are asked to enter some profile information and complete a fall risk assessment. Each assessment consisted of a questionnaire with 12 questions on their clinical falls risk factors and medical history as well as a 30 s standing balance test. An automatic reminder was also set (configurable to a daily, weekly, biweekly or monthly basis) to remind the participant to complete the assessment on a regular basis. Based on their identified level of falls risk, balance impairment and individual fall risk factors, participants are provided with a tailored set of advice and video based exercises, to help them stay healthy and avoid falls. Exercises and advice provided were curated and based on best international practice [26,27].

### 3.1. Questionnaire

Participants were also asked to complete a profile questionnaire on installing the app, which included entering age, gender, height and weight as well whether they use a walking aid, have been diagnosed with Parkinson’s disease or have had a stroke. The questionnaire is based on the American and British Geriatric societies (AGS/BGS) guidelines [28] and aims to capture self-reported data on the main clinical risk factors linked to falls in older adults. The clinical risk factors recorded by the questionnaire included falls history (previous 12 months), polypharmacy, mobility problems, orthostatic hypotension, foot problems, vision problems and the ability to manage routine activities in the home. Falls history was considered in three categories: no falls in the previous 12 months, one fall in the previous 12 months, two or more falls (recurrent faller) in the previous 12 months.

### 3.2. Standing Balance Test

**Figure 1:**
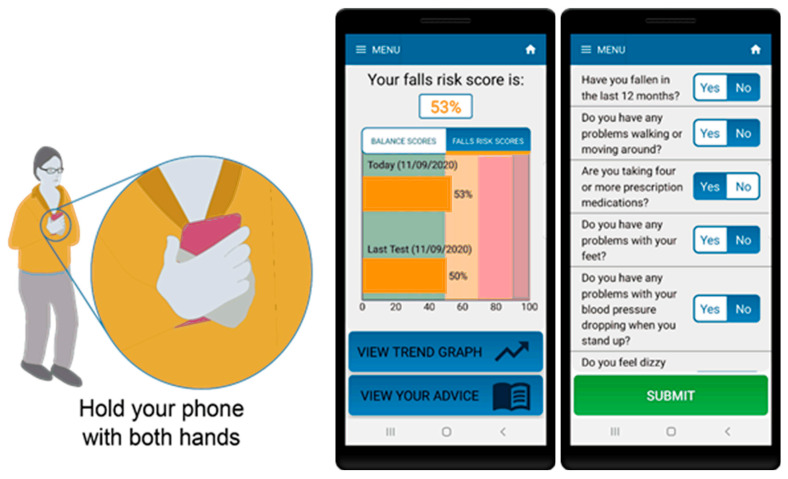
Smartphone app for assessment of balance and falls risk using questionnaire and standing balance test (in semi-tandem stance).

For the standing balance test portion of the assessment, participants were instructed to hold the phone firmly against their torso and stand with their eyes open, in a semi-tandem stance, for 30 s (see Figure 1). The app contains audio and visual instructions on how to prepare for and complete the test as well as an audio cue to indicate test start and stop. The algorithm includes data quality checks to ensure tests are performed correctly including: checking phone is oriented correctly during the test, removing signal artefact resulting from movements due to changes in initial positioning of the phone as well as detection and removal of unusual behaviour or phone movement during the test.

In order to quantify the participant’s balance during the balance test portion of the assessment, a number of features were calculated from the inertial sensor embedded within the smartphone. Smartphone data were sampled at the maximum rate allowed by operating system. To ensure data were sampled evenly, data were interpolated to a 100 Hz sampling rate using linear interpolation. Accelerometer and gyroscope data from the inertial sensor were band-pass filtered between 0.1–5 Hz and calibrated to produce acceleration and angular velocity vectors [29]. To allow for settling at the start of each test, the first and last five seconds of each test were removed.

Nineteen quantitative balance features were calculated for each balance assessment including a balance score (a percentile based metric calculated by comparison against the reference data set) [23] and a fall risk estimate (*FRE_combined_*, a statistical prediction of falls derived using a combination of inertial sensor and questionnaire data, previously validated using a sample of community dwelling older adults [24,30]). *FRE_combined_* uses a classifier fusion approach to combine two logistic regression classifiers, one each based on the questionnaire and inertial sensor data [23,24,25]. The remaining balance features were derived from the smartphone inertial sensor using a previously reported method [25] (for the reference data set, inertial sensor features were calculated from a lumbar mounted inertial sensor using the same method). The RMS amplitude of the *X*, *Y* and *Z*-axis acceleration and angular velocity were used to quantify postural sway in each direction along with the resultant value across the three axes. The frequency domain variability of the signals obtained by the inertial sensor was also examined for both acceleration and angular velocity signals; using the spectral edge frequency (SEF), defined as the frequency below which 95% of the power spectrum of the signal is contained, and the median frequency, defined as the frequency below which 50% of the power spectrum is contained [20]. The spectral entropy (H), a measure of signal complexity [31] of the accelerometer and angular velocity signals, was also calculated.

### 3.3. Statistical Analysis

Due to the unsupervised nature of the standing balance test, large outliers and artefact were apparent in the data. Outliers greater than µ ± 2σ (where µ is sample mean and σ is sample standard deviation) were assumed to be due to unintended movement and removed from the inertial sensor data, while missing data were excluded from analysis.

Inertial sensor and demographic data obtained using the smartphone were compared against the reference data set, which were obtained under clinical conditions, using a z-test with ⍺ = 0.05, by averaging all assessments for each unique participant.

*FRE_combined_* is essentially a posterior probability and is defined as low risk if less than 50%, 50–69% is considered medium risk, while high risk is greater than 70%. The Balance score is percentile based, a Balance score of less than 50% is below the median (50th percentile) and considered low risk, while a score of 50–69% is deemed medium risk. If the balance score for a given category is above 70% (70th percentile) the participant is considered to display balance impairment. These risk category definitions are based on empirically derived thresholds obtained from previous studies [6].

A one-way analysis of variance (ANOVA) was used to test the association between self-reported falls history and both the Balance score and *FRE_combined_*, with ⍺ = 0.05.

Classification of the calculated risk measures according to self-reported falls history was considered as both a two class and a three class problem. Classification performance was evaluated using classification accuracy, sensitivity and predictivity; classification accuracy, is defined as the percentage of participants correctly classified by the algorithm as being a low, medium or high risk. For the 3-class case, a person with no reported falls is considered a non-faller and in the low risk category, a person with one reported fall in the past 12 months is considered a medium risk faller, while a recurrent faller (two or more falls in the past 12 months) is considered high risk. For the 2-class case, a non-faller is defined as a person with no reported falls in the past 12 months (low risk), while a faller is a person with one or more reported falls in the past 12 months (high risk). Sensitivity (Sens) is defined as the percentage of each class (e.g., non-faller, one falls in the past 12 months or recurrent faller) classified correctly (e.g., as low, medium or high risk). Predictivity (Pred) is defined as the proportion of participants, classified as a given class by the algorithm, who were classified correctly.

## 4. Results

A sample of 594 smartphone assessments from 147 unique phones were included in the analysis. The sample contained 113 distinct models of Android smartphone. On average there were 4.0 ± 7.1 assessments per phone.

**Figure 2:**
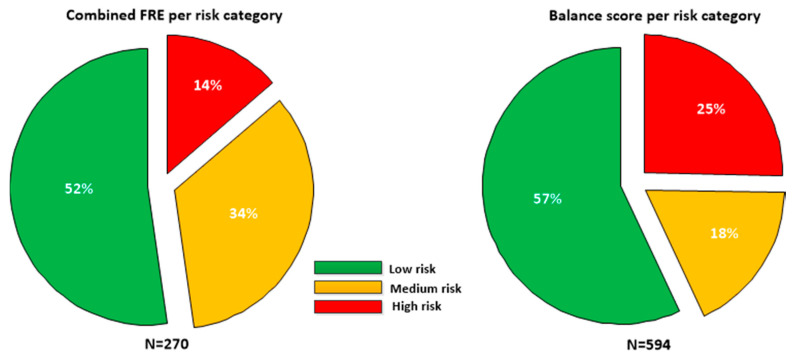
Falls risk estimate (*FRE_combined_*, N = 270) and balance scores (N = 594) broken down in low, medium and high risk categories for the sample. As the FRE is only defined for patients greater than 60, a smaller sample was available than for the Balance score which is defined for all age groups.

Taking each unique phone (as defined by Wi-Fi MAC address) as a surrogate for a single participant (assessments for a unique phone where gender or age were found to have changed were excluded), the sample contained 76 males and 71 females, with a mean age of 56.5 ± 17.0 years, with mean height 167.4 cm and mean weight of 78.2 kg. The falls risk estimate (*FRE_combined_*) was only defined for participants greater than or equal to 60 years of age; there were 270 assessments for participants aged 60 or over. Figure 2 details the proportion of participants in each risk category for each feature.

### 4.1. Inertial Sensor Features

We compared the features calculated from the smartphone inertial sensor data against the reference lumbar sensor data. This is important to establish the validity of the data, to see how values obtained from unsupervised assessments relate to the expected ranges found in the reference data. If the reported measures from the smartphone are out of the expected range for data obtained from a lumbar mounted sensor, this could indicate issues with measurement validity.

Comparison of each of the features calculated from the smartphone inertial sensor data against the reference data using a z-test, found significant differences in each of the features (*p* < 0.05). In addition, comparisons of age, height and weight for the sample against the reference data set found statistically significant differences (*p* < 0.05) for each. Table 1 details the mean and standard deviations for each of the inertial sensor features calculated from the smartphone sensor and lumbar sensor data sets.

**Figure 3:**
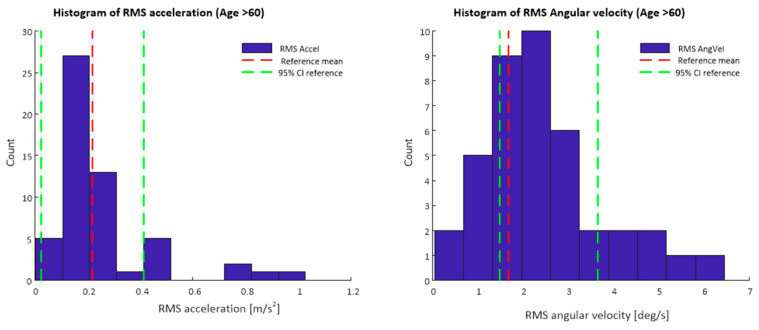
Histograms of RMS acceleration and RMS angular velocity values from smartphone inertial sensor data. Mean values and 95% confidence intervals for the reference data set are also marked.

Figure 3 depicts a histogram of the RMS resultant acceleration and RMS resultant angular velocity values for data obtained from the smartphone app during the 30 s standing balance test Data displayed are only for participants of 60 years of age and older, mean values and confidence intervals for the reference lumbar sensor data set are also shown.

### 4.2. Questionnaire Results

**Figure 4:**
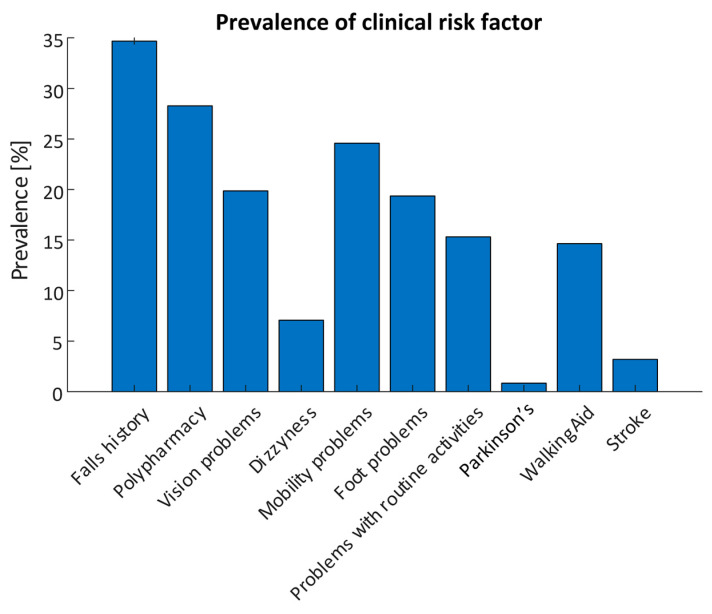
Prevalence of self-reported clinical risk factors obtained using the smartphone app for all assessments (N = 594).

Each assessment contained a questionnaire on clinical risk factors; 594 questionnaire responses were included in the analysis. 206 responses reported a history of falls with a total of 852 recorded falls and 134 recurrent fallers. 168 (28.3%) participants reported polypharmacy (taking four or more prescription medications), while 146 responses reported mobility problems (24.6%). Vision problems were reported in 118 responses (19.9%), Orthostatic Hypotension (OH) was reported in 42 responses (7.1%), while 91 reported changes in their ability to manage routine activities in the home (15.3%). 87 responses (14.6%) reported use of a mobility aid, while 5 (0.8%) reported having Parkinson’s Disease and 19 (3.2%) reported having previously had a stroke. The prevalence of each risk factor are detailed in Figure 4.

**Figure 5:**
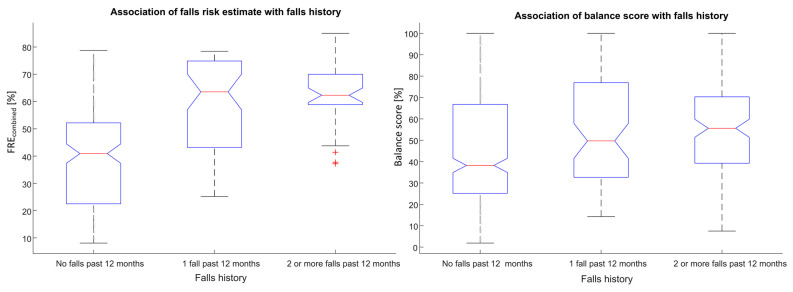
Associations between falls risk (*FRE_combined_*) and Balance scores with self-reported falls history for unsupervised assessments obtained using a smartphone.

A strong association was observed between the calculated falls risk estimate (*FRE_combined_*) and self-reported falls history (F = 60.26, *p* < 0.0001). A strong association was also observed between the calculated Balance score and self-reported falls history (F = 7.45, *p* < 0.001), see Figure 5.

In addition, a strong association of Balance score with self-reported dizziness and Polypharmacy was also found (F = 11.51, *p* < 0.001; F = 13.79, *p* < 0.001 respectively). Balance score and *FRE_combined_* were both also strongly associated with self-reported mobility problems (F = 30.81, *p* < 0.0001; F = 56.91, *p* < 0.0001).

Only 2.4% of participants who reported no falls in the past 12 months (N = 170) were found to have a high risk of falls, while 9.3% of recurrent fallers (N = 43) were found to have a low risk of falls.

When the *FRE_combined_* was used to classify participants into three falls history classes the classification accuracy was 61.8%, with a class sensitivity of 86.9%, 25.0% and 9.0% for the non-faller, 1 fall in the past 12 months and recurrent faller classes respectively. Similarly, if the Balance score was used to classify participants according to falls history the classification accuracy was 54.9% with class sensitivities of 71.6%, 13.9% and 28.4% non-faller, 1 fall in the past 12 months and recurrent faller classes respectively.

When two falls history classes were used, the classification accuracy was 69.9%, while the class sensitivities were 86.9% and 37.9% for the non-faller and faller classes respectively.

Similarly when two falls history classes were used to classify the Balance score, the classification accuracy was 59.9%, while the class sensitivities were 62.9% and 54.4% for the non-faller and faller classes respectively. Classification results are tabulated in Table 2.

## 5. Discussion

This study reports results for the real-world deployment of a smartphone-based machine learning algorithm for the prediction and prevention of falls.

A number of studies have reported smartphone apps for assessment of falls risk and balance. Rasche et al. [32] reported the *Aachen fall prevention* app (APFA), tested on a sample of 79 older adults and subsequently evaluated on a sample of 259 patients [16]. The APFA incorporated a self-reported 10 s postural sway test and a questionnaire, providing a falls risk assessment determined using a set of empirical rules. Hseih et al. [33] investigated the concurrent validity of their app in assessing postural sway when compared against a force-plate and the Physiological Profile Assessment (PPA), in a sample of 30 older adults [21]. Fiems et al. [22] reported on the prospective validity of the *Sway* mobile app, in using postural sway measures to predict falls in a sample of 59 patients with Parkinson’s disease. Papi et al. [34] studied the use of the *Nymbl* app to deliver balance training for 35 participants, measuring improvement using a physical activity questionnaire. The deployment of technologies enabling clinically valid, unsupervised patient-led data capture remains a challenge [19] that requires further investigation.

To our knowledge, the present study is the largest to date and the only study that reports the performance of algorithms that were trained prior to deployment in the app on an independent data set. The algorithms employed here have been previously validated and shown to be valid and accurate in assessing balance and falls risk [6,24,25,30].

Statistically significant differences were observed between quantitative balance features obtained using the smartphone data and those from the reference set. These significant differences in demographic and inertial sensor features persisted even when only those participants (from the smartphone data set), aged 60 or above, were included in the analysis in an attempt to age-match the samples. Differences in clinical measures (such as age, height, weight) suggest that the differences may arise from fundamental demographic differences between the two populations rather than systematic errors in measurement, given that the reported features from the smartphone are in the expected range for data obtained from a lumbar mounted sensor. However, further study on the concurrent validity of this approach is ongoing, in order to validate the use of a smartphone against a lumbar sensor, for measurement of standing balance.

The reported data have limitations which we have attempted to address; data are from diverse participants in real-world scenarios and so were not controlled research-grade data; data were community-sourced using a freely available smartphone app with no restrictions placed on access. As such, some data were missing or were noisy, however, we do not believe there was a systematic or consistent reason for missing data that could affect the results. All users were provided with instructions on test protocol, but we have no way to evaluate treatment fidelity unsupervised in the home. We attempted to address this by performing outlier and artifact rejection on the data as well as through data quality checks built into the app.

In addition, all questionnaire data were self-reported. Due to the nature of the sample population, self-reported data on falls, particularly using retrospective falls history, can be unreliable and under-report the incidence of falls [35]. While 12 month falls history is a strong predictor of future falls, guidelines suggest it is not the most appropriate outcome measure for studies of falls. Future work will consider prospective validation of the reported measures using daily falls diaries or other best practice methods. In addition usability studies, conducted with older adults using the app, are currently ongoing.

The proportions of the sample reported as high, medium and low risk for both *FRE_combined_* and Balance score were slightly different than expected with a lower proportion of participants predicted to be at high risk of falls and balance impairment, suggesting the sample population were healthier than the reference data used to train the algorithms. Statistically significant associations were found between self-reported outcome measures (including falls history, polypharmacy and mobility problems) and the calculated falls risk estimate and Balance scores. This demonstrates the potential value of these metrics in expanding Patient Reported Outcome Measures (PROMs) to include self-directed functional tests. These results suggest that algorithms embedded in a smartphone application using inertial sensor data from a self-directed standing balance test as well as self-reported questionnaire data may produce clinically useful measures of falls risk and balance impairment suitable for use by an older adult, unsupervised in the home environment. Results show a strong association between the smartphone falls risk estimate and balance score with falls history and demonstrates that the proportion of participants at high risk of falls and balance impairment is in line with results expected from a clinical environment. Furthermore, classification results reported are in line with (or exceed) those reported for standard clinical measures such as questionnaire based tools [16,36], the Berg Balance scale [16,37] and the Timed Up and Go test [38,39].

Smartphone apps for assessment of balance and falls risk could be used as part of remote monitoring platform for remote functional assessment or a decentralised clinical trial, they could also be used as part of a comprehensive digital platform to prevent falls, used by older adults and their carers to evaluate risk of falling, prescribe personalised falls prevention programmes with home-based interventions and monitor progress against personalised exercise goals. Such a comprehensive solution would support the needs of healthcare systems and care providers in delivering more targeted digitally-enabled falls prevention and remote care initiatives.

## Figures and Tables

**Table 1:** Comparison of inertial sensors features obtained from a smartphone held against the torso to a statistically independent test set obtained from a lumbar mounted inertial sensor (reference data set).

Inertial Sensor Feature	Smartphone	Lumbar
RMS acceleration (m/s^2^) *	0.24 ± 0.18	0.22 ± 0.10
RMS acceleration-*X*-axis	0.10 ± 0.07	0.10 ± 0.05
RMS acceleration-*Y*-axis (m/s^2^) *	0.07 ± 0.06	0.09 ± 0.04
RMS acceleration-*Z*-axis (m/s^2^) *	0.17 ± 0.16	0.10 ± 0.04
RMS angular velocity (°/s) *	2.20 ± 1.20	1.68 ± 1.01
Median frequency acceleration (Hz) *	2.23 ± 1.47	5.32 ± 1.72
RMS angular velocity-*X*-axis (°/s)	1.29 ± 0.75	0.91 ± 0.56
RMS angular velocity-*Y*-axis (°/s)*	1.17 ± 0.79	0.72 ± 0.43
Spectral edge frequency acceleration (Hz) *	2.79 ± 0.95	4.14 ± 0.46
Spectral entropy acceleration *	0.47 ± 0.13	0.76 ± 0.07
Median frequency angular velocity (Hz) *	2.23 ± 1.40	2.87 ± 1.17
Spectral edge frequency angular velocity (Hz) *	3.45 ± 0.87	3.64 ± 0.50
Spectral entropy angular velocity *	0.5 ± 0.12	0.75 ± 0.06
Age (years) *	52.57 ± 16.89	74.45 ± 6.70
Height (cm) *	170.45 ± 10.81	164.35 ± 9.22
Weight (kg) *	82.56 ± 30.52	74.57 ± 13.69

* Statistically significant differences, obtained using a z-test.

**Table 2:** Classification performance results for classifying *FRE_combined_* and balance score according to falls history. Results are reported for both binary and 3-class falls history. Accuracy (Acc), Sensitivity (Sens) and Predictivity (Pred) are reported.

	Class	*FRE_combined_*	Balance Score		Class	*FRE_combined_*	Balance Score
**Acc (%)**		61.78	54.88	**Acc (%)**		69.87	59.93
**Sens (%)**	*Non-faller*	86.86	71.65	**Sens (%)**	*Non-faller*	86.86	62.89
	*1-fall*	25.00	13.89		*Faller*	37.86	54.37
	*Recurrent faller*	8.96	28.36	**Pred (%)**	*Non-faller*	72.47	72.19
**Pred (%)**	*Non-faller*	72.47	67.80		*Faller*	60.47	43.75
	*1-fall*	19.57	29.41				
	*Recurrent faller*	32.43	25.33				

## Data Availability

The data that support the findings of this study are available from Kinesis Health Technologies Ltd. Restrictions apply to the availability of these data, which were used under license for the current study, and so are not publicly available. Data are however available from the authors upon reasonable request and with permission of Kinesis Health Technologies Ltd, Dublin, Ireland.

## References

[1] World Health Organization (WHO) (2007). WHO global report on falls prevention in older age. WHO Department of Ageing and Life Course.

[2] Burns E.R., Stevens J.A., Lee R. (2016). The direct costs of fatal and non-fatal falls among older adults—United States. J. Saf. Res..

[3] Hartholt K.A., van Beeck E.F., Polinder S., van der Velde N., van Lieshout E.M.M., Panneman M.J.M., van der Cammen T.J.M., Patka P. (2011). Societal Consequences of Falls in the Older Population: Injuries, Healthcare Costs, and Long-Term Reduced Quality of Life. J. Trauma Inj. Infect. Crit. Care.

[4] Sherrington C., Fairhall N.J., Wallbank G.K., Tiedemann A., Michaleff Z.A., Howard K., Clemson L., Hopewell S., Lamb S.E. (2019). Exercise For Preventing Falls in Older People Living in the Community. Cochrane Database Syst. Rev..

[5] Gillespie L.D., Robertson M.C., Gillespie W.J., Sherrington C., Gates S., Clemson L.M., E Lamb S. (2012). Interventions for preventing falls in older people living in the community. Cochrane Database Syst. Rev..

[6] Greene B.R., McManus K., Redmond S.J., Caulfield B., Quinn C.C. (2019). Digital assessment of falls risk, frailty, and mobility impairment using wearable sensors. NPJ Digit. Med..

[7] Cheng W.-Y., Bourke A.K., Lipsmeier F., Bernasconi C., Belachew S., Gossens C., Graves J.S., Montalban X., Lindemann M. (2021). U-turn speed is a valid and reliable smartphone-based measure of multiple sclerosis-related gait and balance impairment. Gait Posture.

[8] Boudreaux E.D., Waring M.E., Hayes R.B., Sadasivam R.S., Mullen S., Pagoto S.L. (2014). Evaluating and selecting mobile health apps: Strategies for healthcare providers and healthcare organizations. Transl. Behav. Med..

[9] Akbar S., Coiera E., Magrabi F. (2020). Safety concerns with consumer-facing mobile health applications and their consequences: A scoping review. J. Am. Med. Inform. Assoc..

[10] Pew-Research Center (2021). Survey of U.S. Adults Conducted Jan. 25-Feb. 8, 2021.

[11] Hughes K.J., Salmon N., Galvin R., Casey B., Clifford A.M. (2018). Interventions to improve adherence to exercise therapy for falls prevention in community-dwelling older adults: Systematic review and meta-analysis. Age Ageing.

[12] McGarrigle L., Boulton E., Todd C. (2020). Map the apps: A rapid review of digital approaches to support the engagement of older adults in strength and balance exercises. BMC Geriatr..

[13] Palestra G., Mohamed Rebiai M., Courtial E., Koutsouris D. (2019). Evaluation of a Rehabilitation System for the Elderly in a Day Care Center. Information.

[14] Uzor S., Baillie L. (2019). Recov-R: Evaluation of a Home-Based Tailored Exergame System to Reduce Fall Risk in Seniors. ACM Trans. Comput. Hum. Interact..

[15] Roeing K.L., Hsieh K.L., Sosnoff J.J. (2017). A systematic review of balance and fall risk assessments with mobile phone technology. Arch. Gerontol. Geriatr..

[16] Rasche P., Nitsch V., Rentemeister L., Coburn M., Buecking B., Bliemel C., Bollheimer L.C., Pape H.-C., Knobe M., Rossi A. (2019). The Aachen Falls Prevention Scale: Multi-Study Evaluation and Comparison. JMIR Aging.

[17] Ghislieri M., Gastaldi L., Pastorelli S., Tadano S., Agostini V. (2019). Wearable Inertial Sensors to Assess Standing Balance: A Systematic Review. Sensors.

[18] Moral-Munoz J.A., Esteban-Moreno B., Herrera-Viedma E., Cobo M.J., Pérez I.J. (2018). Smartphone Applications to Perform Body Balance Assessment: A Standardized Review. J. Med. Syst..

[19] Shany T., Redmond S., Marschollek M., Lovell N. (2012). Assessing fall risk using wearable sensors: A practical discussion. Zeitschrift für Gerontologie und Geriatrie.

[20] Doheny E.P., Greene B.R., Foran T., Cunningham C., Fan C.W., Kenny R.A. (2012). Diurnal variations in the outcomes of instrumented gait and quiet standing balance assessments and their association with falls history. Physiol. Meas..

[21] Hsieh K.L., Roach K.L., Wajda D.A., Sosnoff J.J. (2019). Smartphone technology can measure postural stability and discriminate fall risk in older adults. Gait Posture.

[22] Fiems C.L., Miller S.A., Buchanan N., Knowles E., Larson E., Snow R., Moore E.S. (2020). Does a Sway-Based Mobile Application Predict Future Falls in People With Parkinson Disease?. Arch. Phys. Med. Rehabil..

[23] McManus K., Greene B.R., Ader L.G.M., Caulfield B. Single IMU assessment of postural sway and falls risk in older adults. In Proceeding of the IEEE Conference on Biomedical and Health Informatics (BHI) 2021 and the IEEE Conference on Body Sensor Networks (BSN) 2021 (BHI-BSN 2021).

[24] Greene B.R., McManus K., Caulfield B. Automatic fusion of inertial sensors and clinical risk factors for accurate fall risk assessment during balance assessment. Proceedings of the IEEE Biomedical Health Informatics Conference.

[25] Greene B.R., Doheny E.P., Kenny R.A., Caulfield B. (2014). Classification of frailty and falls history using a combination of sensor-based mobility assessments. Physiol. Meas..

[26] Campbell A.J., Robertson M.C., Gardner M.M., Norton R.N., Tilyard M.W., Buchner D.M. (1997). Randomised controlled trial of a general practice programme of home based exercise to prevent falls in elderly women. BMJ.

[27] Skelton D., Dinan S., Campbell M., Rutherford O. (2005). Tailored group exercise (Falls Management Exercise—FaME) reduces falls in community-dwelling older frequent fallers (an RCT). Age Ageing.

[28] American Geriatrics Society, British Geriatrics Society, American Academy of Orthopaedic Surgeons Panel on Falls Prevention (2001). Guideline for the prevention of falls in older persons. J. Am. Geriatr. Soc..

[29] Ferraris F., Grimaldi U., Parvis M. (1995). Procedure for effortless in-field calibration of three-axis rate gyros and accelerometers. Sens. Mater..

[30] Greene B.R., Redmond S.J., Caulfield B. (2017). Fall Risk Assessment Through Automatic Combination of Clinical Fall Risk Factors and Body-Worn Sensor Data. IEEE J. Biomed. Health Inform..

[31] Viertio-Oja H., Maja V., Sarkela M., Talja P., Tenkanen N., Tolvanenlaakso H., Paloheimo M.P.J., Vakkuri A.P., Ylihankala A.M., Merilainen P. (2004). Description of the Entropytm algorithm as applied in the Datex-Ohmeda S/5tm Entropy Module. Acta Anaesthesiol. Scand..

[32] Rasche P.W.V., Mertens A.W., Bröhl C., Theis S., Seinsch T., Wille M., Pape H.-C., Knobe M. (2017). The “Aachen fall prevention App”—A Smartphone application app for the self-assessment of elderly patients at risk for ground level falls. Patient Saf. Surg..

[33] Hsieh K.L., Fanning J.T., A Rogers W., A Wood T., Sosnoff J.J. (2018). A Fall Risk mHealth App for Older Adults: Development and Usability Study. JMIR Aging.

[34] Papi E., Chiou S.-Y., McGregor A.H. (2020). Feasibility and acceptability study on the use of a smartphone application to facilitate balance training in the ageing population. BMJ Open.

[35] Hoffman G.J., Ha J., Alexander N.B., Langa K.M., Tinetti M., Min L.C. (2018). Underreporting of Fall Injuries of Older Adults: Implications for Wellness Visit Fall Risk Screening. J. Am. Geriatr. Soc..

[36] Cattelani L., Cattelani L., Palumbo P., Palmerini L., Bandinelli S., Becker C., Chesani F., Chiari L. (2015). FRAT-up, a Web-based Fall-Risk Assessment Tool for Elderly People Living in the Community. J. Med. Internet Res..

[37] Thorbahn L.D.B., A Newton R. (1996). Use of the Berg Balance Test to Predict Falls in Elderly Persons. Phys. Ther..

[38] Barry E., Galvin R., Keogh C., Horgan F., Fahey T. (2014). Is the Timed Up and Go test a useful predictor of risk of falls in community dwelling older adults: A systematic review and meta- analysis. BMC Geriatr..

[39] Greene B.R., O’Donovan A., Romero-Ortuno R., Cogan L., Ni Scanaill C., Kenny R.A. (2010). Quantitative Falls Risk Assessment Using the Timed Up and Go Test. IEEE Trans. Biomed. Eng..

